# Typhus and Typhoid Fevers

**Published:** 1866-09

**Authors:** 


DUBLIN, July 19, 1866.

TYPHUS AND TYPHOID FEVERS.—At a recent meeting of the Medical Association, Dr. Henry Kennedy brought forward a number of interesting cases, where the symptoms of typhus and typhoid (or enteric) fever were so mixed that it was impossible to say under which disease the patient was laboring. Dr. Kennedy used these cases in support of opinions, long held, and often expressed by him, that typhus and typhoid fevers were only varieties of a disease produced by one common poison; and that the diseases were not specifically different. He also detailed cases supporting the same views, where the typhus and typhoid forms of fever had arisen in different members of the same family at the same time.

